# Zoledronic acid-induced hepatotoxicity relieved after subsequent infusions in a Chinese woman with glucocorticoid-induced osteoporosis

**DOI:** 10.1186/s40001-015-0161-1

**Published:** 2015-08-22

**Authors:** Yan Jiang, Yong Fu, Xiao-ping Xing, Mei Li, Ou Wang, Wei-bo Xia, Xun-wu Meng

**Affiliations:** Department of Endocrinology, Key Laboratory of Endocrinology, Ministry of Health, Peking Union Medical College Hospital, Chinese Academy of Medical Sciences, 100730 Beijing, China

**Keywords:** Adverse effects, Zoledronic acid, Hepatotoxicity, Glucocorticoid-induced osteoporosis

## Abstract

**Background:**

Zoledronic acid (ZOL) is widely used for treatment of glucocorticoid-induced osteoporosis. The most common adverse effects of ZOL treatment are post-dose symptoms. ZOL-induced hepatotoxicity has very rarely been reported.

**Case report:**

Here, we described a 50-year-old Chinese woman who had vertebral fractures and severe back pain after glucocorticoid therapy for Behcet disease for 13 years. Three days after ZOL 5 mg infusion in April 2012, serum alanine aminotransferase (ALT), aspartate aminotransferase (AST) and gamma-glutamyltransferase (GGT) levels increased by 7.7, 4.9 and 3.0 times, respectively, compared with pre-treatment values. Liver protective agents were administered per os. Her hepatic enzyme levels returned to nearly normal range 9 days post-infusion. In the subsequent ZOL infusion with 1 year interval, serum ALT, AST and GGT levels increased slightly after the second infusion and were sustained to be normal after the third infusion. Her post-dose symptoms alleviated in the meantime.

**Conclusions:**

Hepatotoxicity due to ZOL therapy is a rare, but possible adverse effect which may be relieved after subsequent infusions.

## Background

Although glucocorticoids may effectively be used in the management of inflammatory and immune-mediated disorders, persistent use is associated with side effects, such as bone loss and increased fracture risk [1]. Zoledronic acid (ZOL), a bisphosphonate (BP), intravenous 5 mg once per year, has been approved for treatment of glucocorticoid-induced osteoporosis (GIO) [2, 3]. The overall safety of BP treatment is well established. The most common adverse effects of ZOL treatment are post-dose symptoms [4, 5]. BP-induced liver injury is rare. Only a few cases [6–15] have been reported including two cases of ZOL [13, 14]. We report here in detail the case of hepatotoxicity after ZOL treatment, which was relieved after subsequent infusions.

## Case presentation

The diagnosis of glucocorticoid-induced osteoporosis (GIO) was established in a 50-year-old postmenopausal Chinese woman in April 2012 when she first presented to our clinic. She presented with canker sores, fever, abdominal pain, bloody diarrhea and a past medical history of Behcet disease diagnosed in 1999. From then on, she was treated with glucocorticoid, which alleviated her symptoms. In 2010, she began to have mild back pain. She had T7 and L1 vertebral fractures and severe back pain after a fall in February 2012 while taking methylprednisolone 6 mg once per every other day and tripterygium glucoside 20 mg twice per day. She was given calcitrol 0.25 μg and element calcium 600 mg per day, but her back pain did not improve. She also had diabetes for 3 years and received metformin therapy with a plasma HbA1c 6.6 %. She reported no recent viral or bacterial infections. She did not drink or smoke.

The patient’s physical examination was within normal limits, with her weight being 59.5 kg and height 153.5 cm (BMI 25.3 kg/m^2^), except for tenderness in the back. Laboratory examinations showed normal liver and renal functions. Her T score of lumbar spine and femoral neck was −2.8 and −1.6, respectively.

The patient was given zoledronic acid (ZOL) 5 mg treatment on April 12, 2012. At the day of infusion, she experienced a fever of 38.7 °C, myalgia and arthralgia. Acetaminophen 650 mg per os was given once. These symptoms were self-limited, resolving in 3 days without other nonsteroidal anti-inflammatory drugs (NSAIDs) use. Three days after ZOL infusion, serum alanine aminotransferase (ALT), aspartate aminotransferase (AST) and gamma-glutamyltransferase (GGT) levels increased by 7.7, 4.9 and 3.0 times, respectively, compared with pretreatment values. Serologic tests for hepatitis A, B, C and human immunodeficiency virus were negative. Autoimmunity markers were also normal. Urea, creatinine and blood routine remained normal during follow-up. Abdominal ultrasonography revealed fatty liver. Hepatoprotective agents, glucurolactone (100 mg 3/day) and polyene phosphatidylcholine capsules (456 mg 3/day), were administered per os. Nine days post-infusion, the hepatic enzyme levels returned to nearly normal range. In the subsequent second and third ZOL infusion with 1 year interval, the patient co-received glucurolactone (100 mg 3/day) per os. Her ALT, AST and GGT levels increased slightly after the second infusion and was sustained to be normal after the third infusion (Table 1). Her post-dose symptoms alleviated in the meantime without NSAIDs use. The patient obtained significant relief from back pain 6 months after the first infusion and had no new fractures during the 2 years. Her T score of lumbar spine and femoral neck increased to −2.6 and −1.3, respectively, in June 2014.

**Table 1:** Treatment history and the changes of liver biochemical markers

Date	Treatment	ALT(U/L) (5-40)	AST(U/L) (5-37)	GGT(U/L) (10-67)	TB(μmol/L) (5.1-22.2)	DB(μmol/L) (0-8.6)	ALP(U/L) (30-120)	Symptoms
2012-4-12	ZOL infusion (5 mg)	33	15	45	9.8	2.6	77	Fever 38.7 °C, myalgia, arthralgia
2012-4-15	glucurolactone (100 mg 3/d), polyene )	254	73	135	6.3	2.4	–	
2014-4-18	phosphatidylcholine (456 mg 3/d	126	43	112	7.7	2.5	118	
2012-4-21		66	40	-	11.2	4.3	–	
2013-5-7	ZOL infusion (5 mg), glucurolactone (100 mg 3/d)	28	18	30	9.6	2.9	44	Fever 38.0 °C, arthralgia
2013-5-10		70	40	46	4.6	1.6	-	
2013-5-13		48	22	43	6.0	2.1	58	
2014-6-22	ZOL infusion (5 mg), glucurolactone (100 mg 3/d)	24	20	27	7.2	2.4	51	No fever or pain
2014-6-25		31	28	33	5.9	1.6	55	

*ALT* alanine aminotransferase, *AST* aspartate aminotransferase, *GGT* gamma-glutamyltransferase, *TB* total bilirubin, *DB* direct bilirubin, *ALP* alkaline phosphatase

## Discussion

We reported a 50-year-old Chinese GIO woman who had liver injury after ZOL infusion. Only two cases of hepatotoxicity following ZOL infusion have been previously reported until now. A 53-year Caucasian postmenopausal woman treated for Paget’s disease [13] and a 73-year Chinese woman treated for primary osteoporosis [14] reported transient liver injury 1 day and 3 days after ZOL infusion. They both remained asymptomatic without NSAIDs use. Their serum ALT, AST and GGT returned to normal in 7–12 days. After the first ZOL infusion, our patient took acetaminophen 650 mg once to mitigate pyrexia, myalgia and arthralgia. NSAID (e.g., ibuprofen and acetaminophen) administration, even co-prescribed with intravenous infusion of bisphosphonates, was recommended for reducing common post-dose symptoms [4, 5]. Although NSAID use is a potential cause of liver injury [16], the patient took acetaminophen in case of fever in the past without hepatotoxicity. In addition, one-time routine dose of acetaminophen could not explain the liver injury, which indicated that it was an adverse effect of ZOL.

The other eight cases of hepatotoxicity have been previously documented with BP treatment including alendronate [6–10], clodronate [11], risedronate [12] and ibandronate [15]. Within all of the previous ten cases, six of them had concomitant liver or systemic diseases [8–10, 12, 13, 15]. Our patient also had systemic disease and nonalcoholic fatty liver disease (NAFLD). Most of the adverse hepatic effects were mild to moderate, except in one patient with severe cholestatic hepatitis after ibandronate infusion. However, this patient had systemic lupus erythematosus and was taking hydroxychloroquine and atorvastatin at the same time [15]. None of these cases reported the following therapy for osteoporosis. To our knowledge, this is the first case that revealed ZOL-induced hepatotoxicity could be relieved after subsequent infusions. It is suggested that ZOL could be cautiously used again in patients with mild to moderate transient elevation of liver enzymes after the first infusion.

The mechanism by which ZOL may cause liver damage is elusive. It has been reported that the number of patients who had any of the commonly reported post-dose symptoms such as pyrexia, myalgia and arthralgia was high after the first infusion of ZOL treatment, but decreased substantially after subsequent infusions [4]. Post-dose symptoms, especially fever after ZOL administration, are due to increase in serum TNF-alpha and IL-6 [17]. TNF-alpha together with other cytokines mediate and contribute to liver damages [18]. We speculated that cytokines may play a role in the hepatotoxicity of ZOL.

## Conclusions

Hepatotoxicity due to zoledronic acid therapy is a rare, but possible adverse effect which may be relieved after subsequent infusions. We should pay attention to patients with concomitant liver or systemic diseases and monitor the liver biochemical indicators after treatment.

## Consent

Written informed consent was obtained from the patient for publication of this case report. A copy of the written consent is available for review by the Editor-in-Chief of this journal.

## Abbreviations

GIO: glucocorticoid-induced osteoporosis
ZOL: zoledronic acid
BP: bisphosphonate
